# Supplementary material for: Identification of Axinellamines A and B as Anti-Tubercular Agents
Source: Mar Drugs. 2024 Jun 28;22(7):298. doi: 10.3390/md22070298 (PMC11277618; doi:10.3390/md22070298)

Supplementary Materials

Identification of Axinellamines A and B as Anti-Tubercular Agents

Emily J. Strong [1,#], Lendl Tan [1,#], Sasha Hayes [2,3], Hayden Whyte [1], Rohan A. Davis [2,3] and Nicholas P. West [1,*]

[1] School of Chemistry and Molecular Biosciences, and the Australian Infectious Diseases Research Centre, The University of Queensland, Brisbane QLD 4072, Australia
[2] Griffith Institute for Drug Discovery, School of Environment and Science, Griffith University, Brisbane QLD 4111, Australia
[3] NatureBank, Griffith University, Brisbane QLD 4111, Australia
* Correspondence: n.west@uq.edu.au
[#] These authors contributed equally to this work

Contents:

Figure S1 *M. tuberculosis* inhibition of bioassay-guided fractions from NatureBank extracts
Figure S2 ^{1}H NMR (800 MHz) Spectrum of TFA Salt of Axinellamine A (**1**) in DMSO-d_6 at 25 °C
Figure S3 UHPLC-MS of TFA Salt of Axinellamine A (**1**)
Figure S4 ^{1}H NMR (800 MHz) Spectrum of TFA Salt of Axinellamine B (**2**) in DMSO-d_6 at 25 °C
Figure S5 UHPLC-MS of TFA Salt of Axinellamine B (**2**)

Figure S1: *M. tuberculosis* inhibition of bioassay-guided fractions from NatureBank extracts

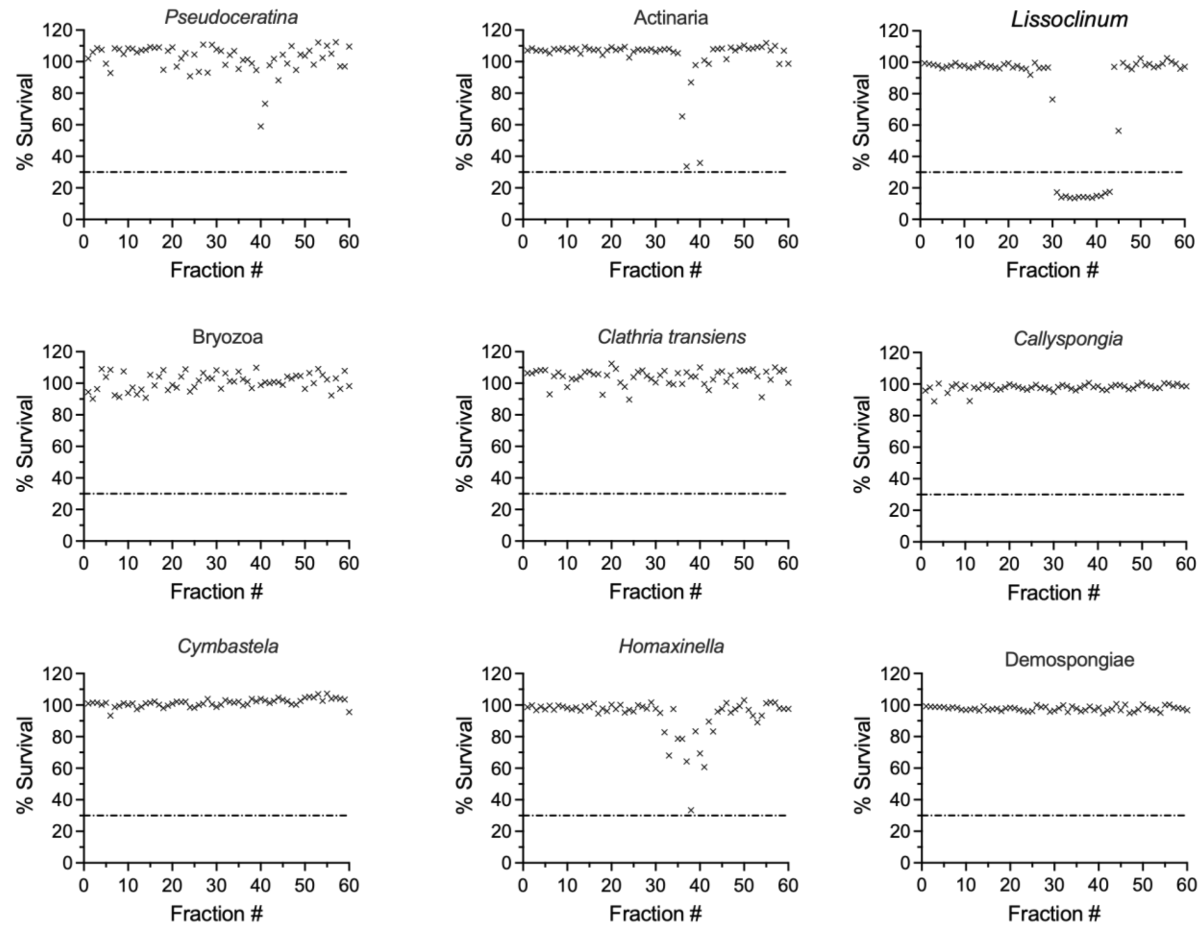

Figure S2: ^{1}H NMR (800 MHz) Spectrum of TFA Salt of Axinellamine A (**1**) in DMSO-d_6 at 25 ^{o}C

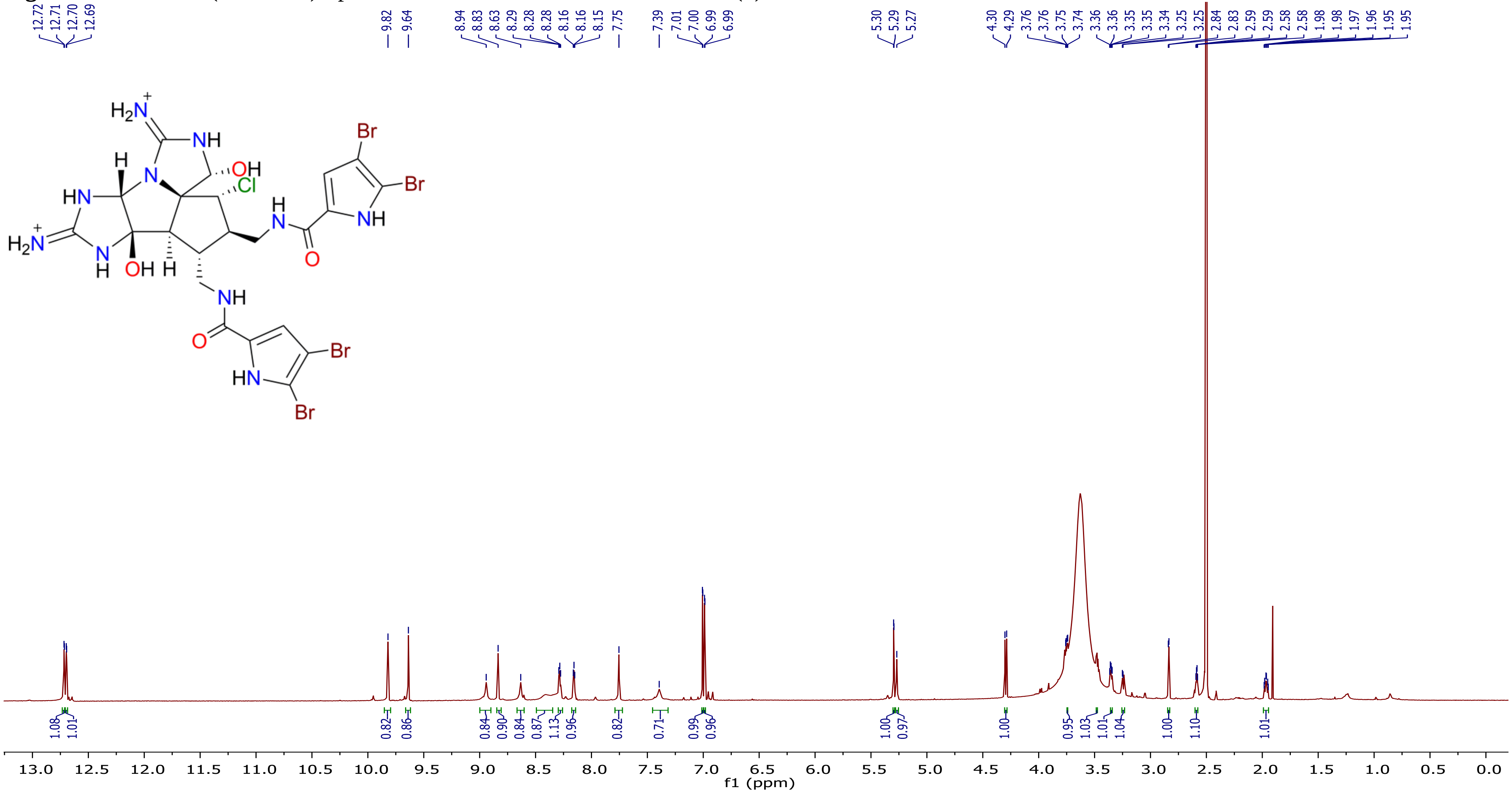

Figure S3: UHPLC-MS of TFA Salt of Axinellamine A (**1**)

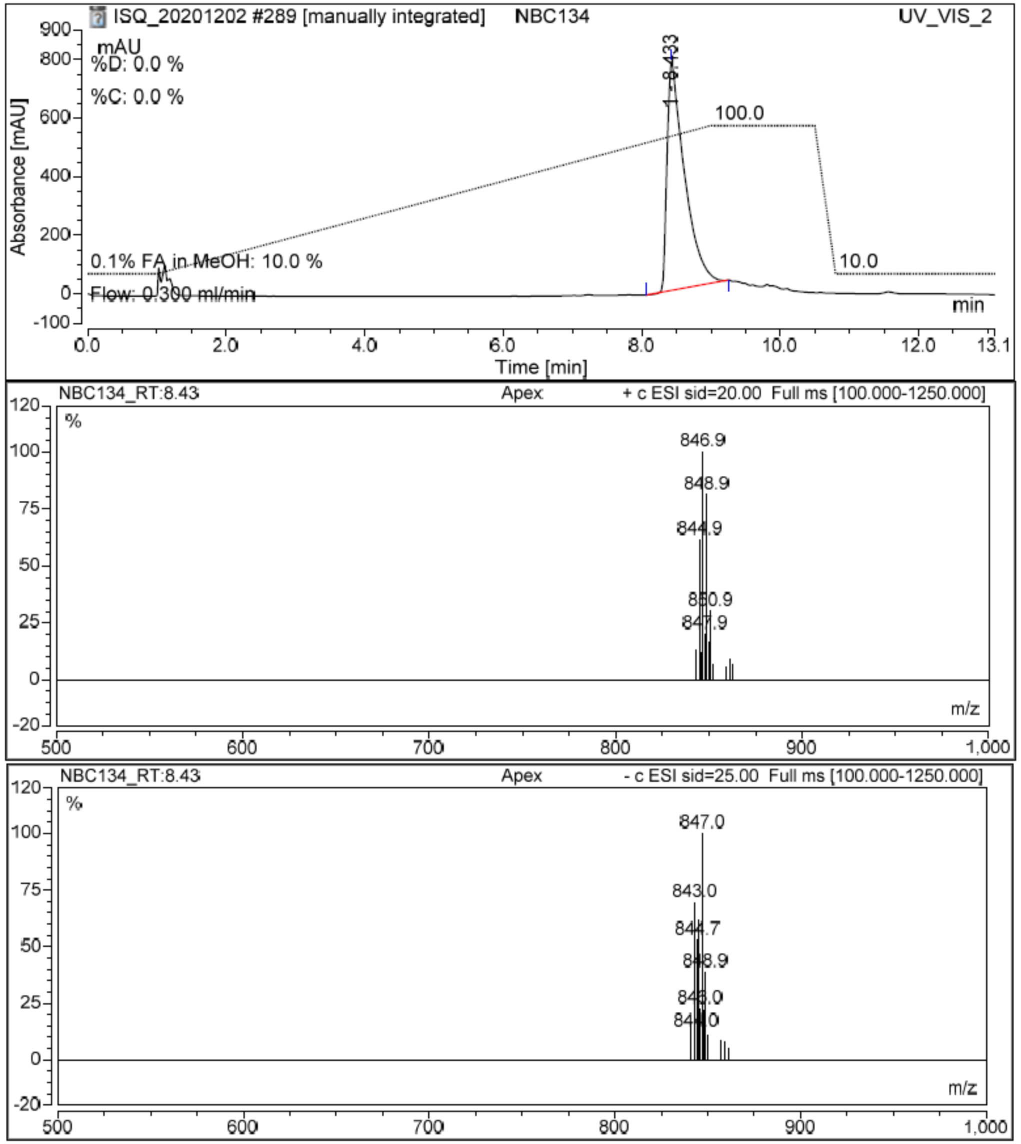

Figure S4: ^{1}H NMR (800 MHz) Spectrum of TFA Salt of Axinellamine B (**2**) in DMSO-d_6 at 25 °C

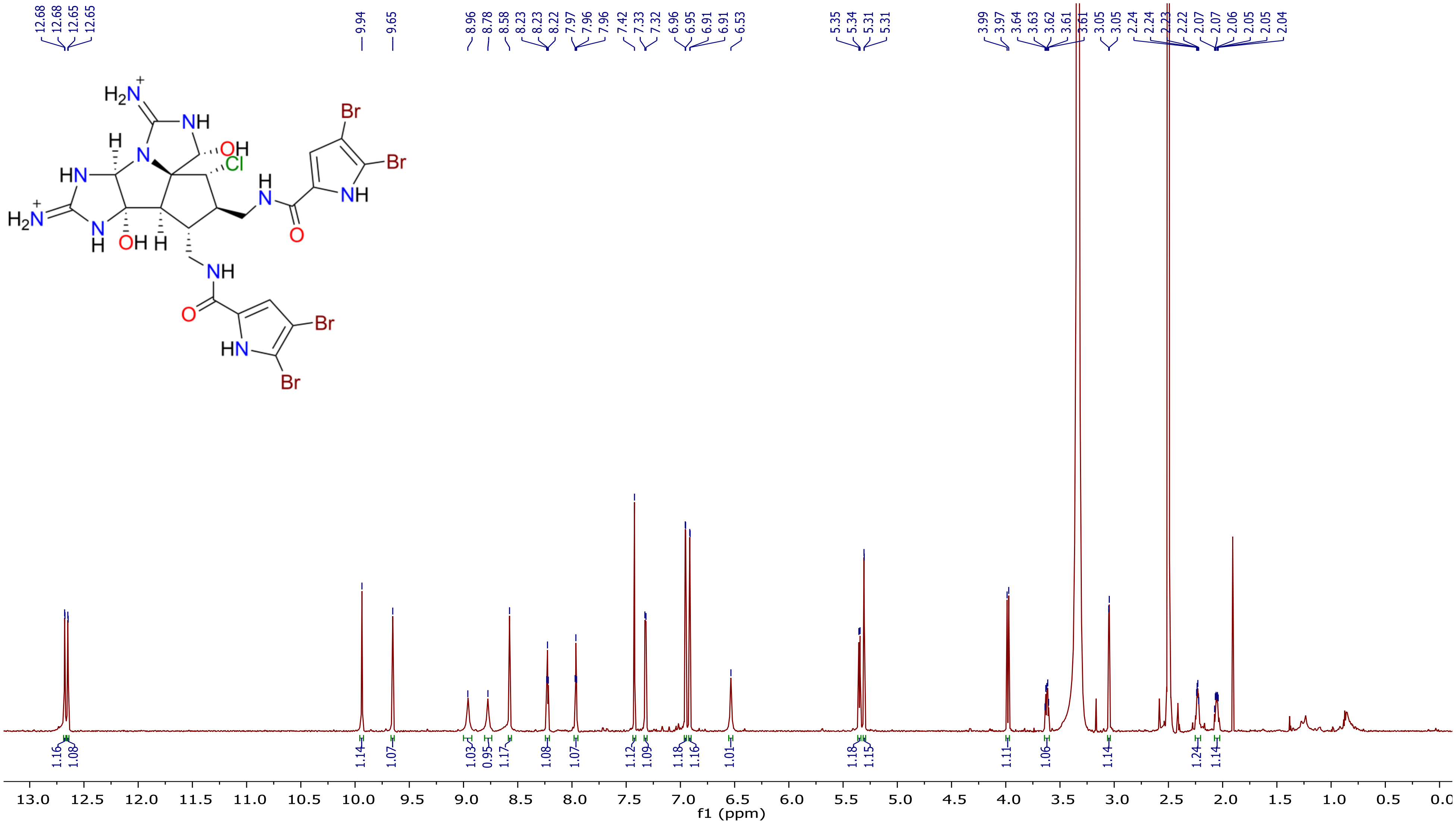

Figure S5: UHPLC-MS of TFA Salt of Axinellamine B (**2**)

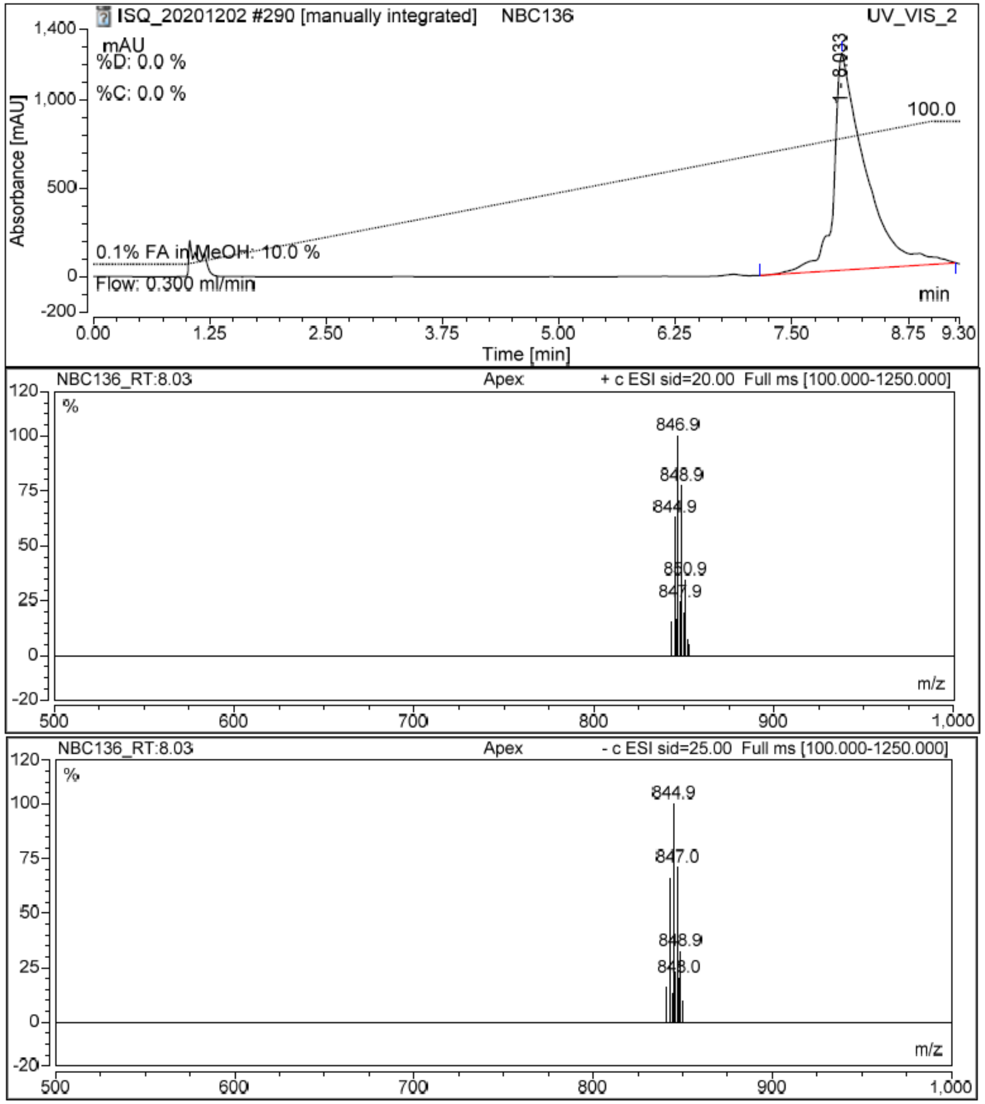

Supplement: Supplementary file 1 [file marinedrugs-22-00298-s001.zip › marinedrugs-3064781-supplementary.pdf]
